# Dual-Layered Approach of Ovine Collagen-Gelatin/Cellulose Hybrid Biomatrix Containing Graphene Oxide-Silver Nanoparticles for Cutaneous Wound Healing: Fabrication, Physicochemical, Cytotoxicity and Antibacterial Characterisation

**DOI:** 10.3390/biomedicines10040816

**Published:** 2022-03-31

**Authors:** Atiqah Salleh, Norlaila Mustafa, Yeit Haan Teow, Mohd Nor Fatimah, Fauzul Azim Khairudin, Ishak Ahmad, Mh Busra Fauzi

**Affiliations:** 1 Centre for Tissue Engineering and Regenerative Medicine, Faculty of Medicine, Universiti Kebangsaan Malaysia, Kuala Lumpur 56000, Malaysia; atqhsalleh@gmail.com; 2 Department of Internal Medicine, Faculty of Medicine, Universiti Kebangsaan Malaysia, Kuala Lumpur 56000, Malaysia; norlaila@ppukm.ukm.edu.my; 3 Department of Chemical and Process Engineering, Faculty of Engineering and Built Environment, Univesiti Kebangsaan Malaysia, Bangi 43600, Malaysia; yh_teow@ukm.edu.my; 4 KPJ Ampang Puteri Specialist Hospital, Suite No. 3-13, No. 1, Jalan Mamanda 9, Taman Dato’ Ahmad Razali, Ampang 68000, Malaysia; drfatimahnor@gmail.com; 5 Faculty of Science and Technology, Universiti Kebangsaan Malaysia, Bangi 43600, Malaysia; fauzul.azim.fa@gmail.com (F.A.K.); gading@ukm.edu.my (I.A.)

**Keywords:** collagen, gelatin, cellulose, bilayer scaffold, GO-AgNP, nanoparticles, wound healing

## Abstract

Tissue engineering products have grown rapidly as an alternative solution available for chronic wound and burn treatment. However, some drawbacks include additional procedures and a lack of antibacterial properties that can impair wound healing, which are issues that need to be tackled effectively for better wound recovery. This study aimed to develop a functionalized dual-layered hybrid biomatrix composed of collagen sponge (bottom layer) to facilitate cell proliferation and adhesion and gelatin/cellulose hydrogel (outer layer) incorporated with graphene oxide and silver nanoparticles (GC-GO/AgNP) to prevent possible external infections post-implantation. The bilayer hybrid scaffold was crosslinked with 0.1% (*w*/*v*) genipin for 6 h followed by advanced freeze-drying technology. Various characterisation parameters were employed to investigate the microstructure, biodegradability, surface wettability, nanoparticles antibacterial activity, mechanical strength, and biocompatibility of the bilayer bioscaffold towards human skin cells. The bilayer bioscaffold exhibited favourable results for wound healing applications as it demonstrated good water uptake (1702.12 ± 161.11%), slow rate of biodegradation (0.13 ± 0.12 mg/h), and reasonable water vapour transmission rate (800.00 ± 65.85 gm^−2^ h^−1^) due to its porosity (84.83 ± 4.48%). The biomatrix was also found to possess hydrophobic properties (48.97 ± 3.68°), ideal for cell attachment and high mechanical strength. Moreover, the hybrid GO-AgNP promoted antibacterial properties via the disk diffusion method. Finally, biomatrix unravelled good cellular compatibility with human dermal fibroblasts (>90%). Therefore, the fabricated bilayer scaffold could be a potential candidate for skin wound healing application.

## 1. Introduction

Tissue engineering is a rapidly developed technology that promotes faster wound healing in chronic wounds, especially skin substitute development. Skin substitutes or bio-templates are potential medical products produced through tissue engineering approaches for an alternative treatment, temporarily or permanently replacing and restoring in order to support tissue loss in chronic wounds [1]. The skin substitutes are classified into two types; acellular and cellular skin substitutes referred to without and with cells. For instance, in the case of cellular skin substitutes, they are made using a specific biotemplate combined with allogeneic or autologous cells either from stem cells or adult skin cells. Transcyte^®^ is an example of cellular skin substitutes commercially available, consisting of porcine type I collagen and nylon mesh seeded with fibroblasts as the bottom layer and silicone membrane as the upper layer. It has been clinically proven to improve the healing rate in burn wounds [2]. However, the materials are significantly expensive and there is a high risk of rejection by the immune system [3]. Meanwhile, acellular skin substitutes are described as alternative skin substitutes, which only consist of functionalized biomaterials. For instance, Integra^®^ is an acellular skin substitute that consists of two layers mimicking the skin structure. The bottom layer consists of bovine collagen type I, while the upper layer consists of a silicone membrane infused with glycosaminoglycan [4]. This type of skin substitute has brought further attention to wound healing research as it has low immunogenicity effects and has received positive feedback from clinical trials [5]. However, a secondary intervention is necessary post-implantation for the silicon removal prior to achieving complete wound recovery. It may contribute to wound healing prolongation, with evidence of potential high risk of secondary infection and causing bacteraemia. Thus, other alternative strategies need to be explored for better future improvement while reaching newly explored precision medicine strategies.

The most well-known biomaterial used in wound healing research is collagen. Collagen is classified into four main types; types I, II, III, and IV. However, the current study used collagen type I as it is abundant in skin tissue and easily extracted from various sources via enzymatic- or chemical-based approaches [6]. The previous study showed that collagen type I was successfully extracted from ovine and provided favourable results in in vitro and in vivo experiments 10 years ago [7]. Collagen type I extraction from ovine tendon demonstrated a high yield and comparable chemical characteristics with the commercialized collagen type I. On the other hand, gelatin is the collagen derivative that contains denatured collagen fibres. It is widely used as a biomaterial as it possesses higher mechanical strength, higher flexibility, and is modifiable compared to collagen. Gelatin is presented as being highly biocompatible towards cells. A combination of gelatin with other biomaterials is highly recommended, as it further increases the stability of gelatin, including its degradation ability and mechanical strength [8,9,10]. Cellulose has also been extensively used in biomaterial development as a suitable material to be hybridized with gelatin. To date, cellulose can be transited to a nano-structure with an optimum size range of 10–100 nm. Nanocellulose can be fabricated into six types: nanofibrils, nanofibers, nanowhiskers, nanocrystals, nanorods, and nanoballs. Nanocellulose has many benefits, including a large surface area that helps increase the adsorption properties, its mechanical strength and flexible morphologies, and because it is biocompatible with a wide range of cells [11].

Currently, researchers worldwide are working on integrating antibacterial compounds into biomaterials that provide a synergistic effect in tissue wound healing, especially for chronic wounds [12]. Various antibacterial components have been studied, including drug-based components, a natural compound, and metal nanoparticles that are also used in wound healing research [13]. The main reason for incorporating metal nanoparticles into biomaterials is to enhance the antibacterial properties of the scaffolds. Silver nanoparticles (AgNP) are widely known for their high antibacterial properties towards both gram-negative and gram-positive bacteria due to their highly variable mechanism of action, including the penetration of silver ions into the bacterial cytoplasm [14,15]. Even though AgNP has high antibacterial properties, it is precarious and very reactive towards the cells. Hence, graphene oxide (GO) was added to help stabilize the AgNP. GO is an oxidised form of graphene derivatives encompassing a hydrophilic functional group [16]. The presence of GO to AgNP stabilised the composition and gave good dispersion of nanomaterials as well [17,18]. GO also has an antibacterial mechanism, which is influenced by the degree of hydrophlicity of the nanoparticles for the bacterial membrane adhesion, and thus inhibits bacterial proliferation. The hybridization of graphene nanoparticles with other metal nanoparticles has also shown high antibacterial activity by disrupting the bacterial membrane, which causes expulsion of bacterial biomolecules such as protein and lipids [19,20]. Thus, hybrid graphene oxide-silver nanoparticles (GO-AgNP) were chosen in the biomaterials as they have highly stable nano-composition and good antibacterial activity.

Based on the current growth and broader comprehensive wound healing dynamic process, an authentic bilayer skin substitute was recently developed with extensive potential in the wound-based markets. The application of bilayer composites has demonstrated better-wound healing efficiency than single layer scaffolds [21,22,23]. In this work, a bilayer collagen hybrid biomatrix, with or without crosslinking, was fabricated to treat the full thickness of skin wounds potentially. The bilayer scaffolds were compared with a single-layer collagen sponge that was established in our research laboratory. The scaffolds’ physicochemical, mechanical, antibacterial, and cell viability were further evaluated. This innovation involved dual approaches for a time post-implantation. Whereas the bottom layer will gradually degrade in the implanted area, the upper layer will peel off together with the crusted wound over time. Therefore, the current study hypothesised that the bilayer scaffold could be a promising candidate for future treatment strategy in wound care management.

## 2. Materials and Methods

This study was approved accordingly by Universiti Kebangsaan Malaysia (UKM) Research Ethics Committee (UKM PPI/111/8/JEP-2020-722), covering all related protocols and sample taking.

### 2.1. Fabrication of Collagen Scaffold

The collagen extraction was performed according to the method described by Fauzi et al. [7]. The ovine tendon was soaked in 0.35 M (*v*/*v*) acetic acid (Merck, Darmstadt, Germany) overnight at 4 °C and was then blended into a collagen solution. In order to obtain pure collagen type I, salting out was performed using sodium chloride (NaCl; Merck, Darmstadt, Germany) and dialyzed accordingly followed by pre-freezing at −80 °C prior to the freeze-drying process. The dried collagen was weighed and dissolved in 0.35 M (*v*/*v*) at 4 °C to achieve a final concentration of 15 mg/mL. The collagen solution was then poured into the desired mould and was pre-frozen at −80 °C for 6 h, followed by freeze-drying (Ilshin, Korea) 24–48 h. The fabricated collagen scaffolds were then crosslinked with 0.1% (*w*/*v*) genipin (GNP; FUJIFILM Wako, Osaka, Japan) and were subsequently freeze-dried for 24–48 h before further analysis. The non-crosslink collagen sponge and crosslinked collagen sponge were labelled NC and CR, respectively. The NC acts as the control of the study as an untreated group that can retain its structure due to the lyophilization process.

### 2.2. Fabrication of Bilayer Scaffold

**Figure 1:**
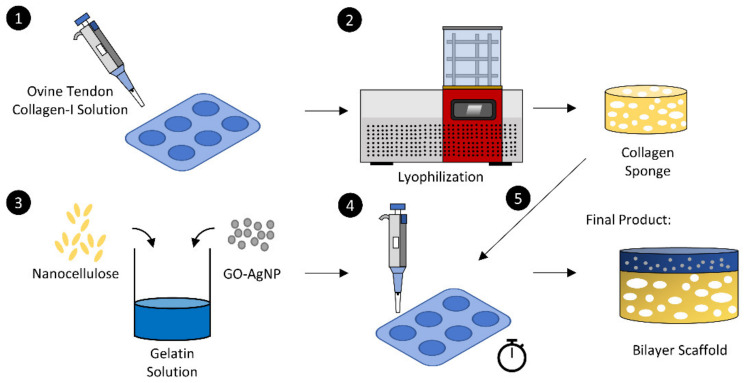
Schematic illustration of the fabrication process of the bilayer scaffolds.

The hydrogel was created using gelatin and nanocellulose natural-based materials. The 0.5% (*w*/*v*) gelatin (Nitta-Gelatin Inc., Osaka, Japan) was prepared in distilled water at 40 °C followed by the addition of nanocellulose powder into the gelatin solution with a final concentration of 0.05% (*w*/*v*). The hydrogel was left at room temperature (22–25 °C) to solidify, and, before the hydrogel was fully polymerized, NC and CR were added to prepare the bilayer scaffold. The scaffold was then frozen at −80 °C for 6 h before freeze-drying for 24–48 h. Figure 1 demonstrates the overall fabrication process of the bilayer scaffold. The non-crosslink bilayer scaffold, crosslinked bilayer scaffolds, and hydrogel were labelled BNC, BCR, and GC, respectively.

### 2.3. Swelling Ratio

The swelling ratio of the bilayer scaffold was measured by immersing the scaffold in phosphate-buffered saline (PBS) at room temperature for different periods. The method was adopted with minor modifications from Khoushabi et al. [24]. The swelling ratio (SR) was determined using the following formula:

SR = [W_f_ − W_i_]/W_i_ × 100,

where W_i_ and W_f_ is the weight of scaffold in dry and hydrated forms, respectively.

### 2.4. In Vitro Biodegradation

The biodegradation rate was determined by enzymatic degradation using 0.0006% collagenase type I solution. The biocomposite scaffold was weighted before immersing into 0.0006% collagenase and was incubated at 37 °C. The rate of the biodegradation (BP) was determined using the following formula:

BP = [W_i_ − W_f_]/Time,

where W_i_; initial weight and W_f_; final Weight

### 2.5. Degree of Crosslinking 

The degree of crosslinking was determined using ninhydrin assay. The glycine standard curve was prepared by serial dilution (0.006, 0.0125, 0.025, 0.05, and 0.1 mg/mL). Then, the bioscaffolds were weighed and immersed in 10× ninhydrin solution (10 mg of the scaffold into 200 µL of ninhydrin solution). The bioscaffolds were boiled at 100 °C for 2 min and were left to cool down. Next, 95% of ethanol was added to dilute the solution. The solution was then placed in a 96-well plate and read under 570 nm absorbance. The degree of crosslinking (DC) was determined using the formula below:

DC = [A_nc_ − A_c_]/A_nc_ × 100,

where A_nc_; absorbance of non-crosslink scaffold and A_c_; absorbance of crosslinked scaffold

### 2.6. Resilience

The resilience of scaffolds is to measure the ability of the scaffolds to retain its original shape after asserting pressure on the scaffolds. A total of 300 g of the metal load was placed on the biocomposite scaffolds for 2 min. Next, the bioscaffolds were immersed in distilled water for 2 min. The area of thickness before compression, after compression, and rehydration were documented and analyzed in ImageJ software (NIH, Bethesda, MD, USA). The resilience (R) was determined using the following formula:

R = A_i_ − A_c_/A_f_ × 100,

where R; the resilience of scaffolds A_i_; area of thickness before compression and A_f_; area of thickness after rehydration, and A_c_, area of thickness after compression.

### 2.7. Contact Angle

The contact angle was done by preparing a biocomposite solution slide. The slides were prepared by smearing the biocomposite solution using a blood smear method. The slides were then left to dry. Five µL of distilled water was slowly placed on the slides. The contact angle of the biocomposite slides and water droplets were recorded. The results were analyzed using ImageJ software (NIH, Bethesda, MD, USA).

### 2.8. Water Vapor Transmission Rate

The water vapour transmission rate (WVTR) was adapted from Rui et al., 2016 and was evaluated according to the American Society for Testing and Materials (ASTM) standard [25,26]. Briefly, the bioscaffold was placed on the top of a glass bottle that contained 10 mL of distilled water. The sample was then placed in a controlled environment as 5% CO_2_ and 37 °C incubator. The results were recorded and analyzed using the formula below:

WVTR = (W_i_ − W_f_)/(A × Time),

where W_i_; initial weight, W_f_; final weight and A; surface area of cylinder bottle.

### 2.9. Microporous Structure Study

The surface topography and cross-section microstructure of composite bioscaffolds were studied using scanning electron microscopy (SEM) at 15 kV. The composite bioscaffold is fixed with 3% glutaraldehyde and undergoes serial ethanol dehydration. The scaffolds were freeze-dried overnight, followed by nanogold-coating prior to SEM observation. The pore size of the scaffolds was measured randomly using measurement software. Field emission SEM was used to observe the fibrous structure under higher magnification. The porosity test was performed according to the liquid displacement method using ethanol. The data were analyzed using the formula below:

Porosity = [(W_f_ − W_i_)/ρV] × 100,

where W_f_, final weight, W_i_, initial weight, ρ density of ethanol and V, the volume of the scaffold.

### 2.10. Energy Dispersive X-ray Spectroscopy

The elemental contents on the composite bioscaffolds’ surface were measured by energy dispersive X-ray microanalysis (EDX) (Phenom, Eindhoven, The Netherlands). NC was used as a control.

### 2.11. Fourier-Transform Infrared Spectroscopy

Fourier transform infrared (FTIR) spectroscopy was utilized to characterize the composite bioscaffolds (PerkinElmer, Waltham, MA, USA). The FTIR spectra have been obtained from a portion of composite flakes using the FTIR spectrophotometer. Measurement was conducted at wavelength range of 4000–500 cm^−1^ at a resolution of 2 cm^−1^ per point at room temperature.

### 2.12. Mechanical Properties Analysis

The mechanical properties of the composite bioscaffolds were evaluated using a tensile testing analyzer (Instron, Norwood, MA, USA). For tensile strength measurements, the cylindrical samples used were approximately 10 mm in diameter and 1 mm in height in accordance with the sample holder. The average data of six samples were recorded, and the values are expressed as means ± standard error.

Simple compression test was done to evaluate the ability of scaffolds to withstand force (compressive load). The total load applied on the fabricated scaffolds is 3 N. The samples used were approximately 16.4 mm in diameter and 4 mm in height. The compression of modulus (E) was obtained using the formula below [27,28]:

E = σ/ε,

σ = Compressive force per unit area (stress),

ε = Changes in volume per unit volume (strain),

where σ is the compressive stress and ε is the strain.

### 2.13. X-ray Powder Diffraction

At room temperature, the XRD pattern of scaffolds was examined using a Bruker D8 Advance X-ray diffractometer equipment (Bruker AXS GmbH, Karlsruhe, Germany). The radiation wavelength was 0.154 nm, and a current of 25 mA was supplied to the samples for measurement from a broad focus Cu tube operating at a voltage of 40 kV. A 2-theta vs. intensity (a.u.) chart was used to collect the data.

### 2.14. Disk Diffusion

According to the National Committee for Clinical Laboratory Standards [29], the antibacterial activity of nanoparticles was performed through an agar diffusion assay. Using a sterile cotton swab, the bacterial suspension was spread on the Mueller-Hinton (Sigma-Aldrich, Burlington, MA, USA) agar. Then, different concentrations of nanoparticles (0.1, 0.05, 0.025, 0.0125 and 0.00625 mg/mL) were placed on the agar. The GO-AgNP was obtained from our collaborator and was produced according to the procedure reported by Mahmoudi et al. [30]. Briefly, the AgNP were decorated onto the GO nanoplate using chemical reduction method. The hybrid nanoparticles are spherical with a range of size between 2 and 5 nm. The plates were incubated at 37 °C overnight. The diameter of the zone of inhibition was recorded.

### 2.15. Cell Isolation and Culture

The protocol was adapted from the method by Seet et al., 2012 [31]. The redundant skin samples were obtained from all consenting healthy patients undergoing abdominoplasty or facelift surgery. In brief, skin samples (3 cm^2^) were cleaned from unwanted fragments such as fat, hair, and debris, and minced into small pieces (approximately 2 mm^2^). The skin was digested in 0.6% collagenase type I (Worthington, Lakewood, NJ, USA) for 5–6 h in a 37 °C incubator shaker followed by cell dissociation using 0.05% Trypsin-EDTA (Gibco, Carlsbad, CA, USA) for 8–10 min. The human dermal fibroblasts were obtained and culture in fibroblasts growth medium (F-12: Dulbecco’s modified eagle medium (Gibco, Carlsbad, CA, USA) supplemented with 10% fetal bovine serum (FBS) (Gibco, Carlsbad, CA, USA).

### 2.16. Live & Dead Assay

A live-dead viability kit (Invitrogen, Waltham, MA, USA) was used to determine cell viability at 24 h after being exposed to treatment. The cells were seeded on the scaffolds and incubated for 24 h. Cell seeded composite bioscaffolds were rinsed with sterile PBS and incubated with a mixture of calcein-AM and ethidium homodimer-1 according to the manufacturer’s instructions. The cells were visualized using a Nikon Eclipse Ti fluorescence microscope (Nikon, Tokyo, Japan).

### 2.17. Statistical Analysis 

The data obtained were analyzed using GraphPad Prism version 8.0 (GraphPad Software, Inc., San Diego, CA, USA). The data was presented in the form of mean ± standard deviation (SD) and collected from all parameters. One-way and two-way analysis of variance (ANOVA) was applied to compare the control and the treatment groups. The data were considered as significant difference when the *p*-value is <0.05.

## 3. Results

### 3.1. Physical Properties of the Fabricated Bioscaffold

#### 3.1.1. Swelling Ratio

**Figure 2:**
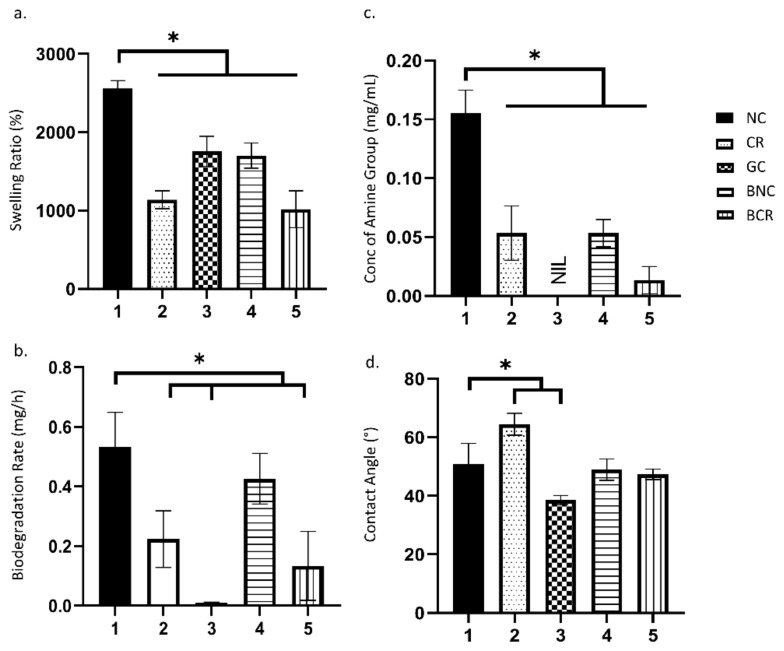
The physical characterisation of the scaffolds at 37 °C. (**a**) the water absorption ability, (**b**) biodegradation rate, (**c**) degree of crosslinking, and (**d**) the contact angle. * *p* < 0.05 indicates the siginificant differences of the fabricated scaffolds.

The swelling ratio represents the ability of the scaffolds to absorb water, which is essential to absorb excessive exudates from the wound site. The swelling ratio of the scaffolds is described in Figure 2a. All fabricated bioscaffolds demonstrated a higher swelling ratio of more than 1000% (acceptable baseline) [32]. The non-crosslinked collagen sponge (NC) showed the highest water absorption ability with 2559.83 ± 99.84% followed by gelatin/nanocellulose hydrogel (GC) with 1757.22 ± 192.28%. The fabricated bilayer bioscaffolds demonstrated significantly lower swelling ratios compared to a single-layer collagen sponge where the non-crosslinked bilayer scaffold (BNC) and crosslinked bilayer scaffold (BCR) exhibited swelling ratios of 1702.12 ± 161.11% and 1017.61 ± 235.47%, respectively.

#### 3.1.2. Biodegradation

The biodegradation rates of the scaffolds were evaluated using the enzymatic degradation approach, as shown in Figure 2b. The NC was able to degrade faster (0.43 ± 0.08 mg/h) compared to crosslinked collagen sponge (CR) (0.22 ± 0.10 mg/h). The addition of genipin was able to decrease the degradation rate in the scaffolds. Additionally, the cellulose hydrogel layer was found to significantly help in decreasing the degradation rates, especially in BCR (0.13 ± 0.12 mg/h). The addition of cellulose in the hydrogel helps in slowing down the degradation process (0.001 ± 0.001 mg/h).

#### 3.1.3. Crosslinking Degree

The degree of crosslinking was determined by quantifying the free amine group via ninhydrin assay (Figure 2c). Most of the crosslinked scaffolds presented a lower free amine concentration than non-crosslink scaffolds. There was a significant decrease (*p* < 0.05) in the concentration of the free amine group in the NC (0.16 ± 0.02 mg/mL) with post-crosslinked with 0.1% genipin (0.05 ± 0.02 mg/mL). The BNC (0.05 ± 0.01 mg/mL) also exhibited a slightly decreased free amine group after crosslinking (0.01 ± 0.01 mg/mL). Overall, genipin is a good crosslinking agent for the scaffolds as the concentration of the free amine group decreases after crosslink. The bilayer scaffolds were found to have a lower concentration of amine group as the free amine group was occupied by the upper layer of the scaffolds, which block the access of ninhydrin to bind to amine group of scaffolds.

#### 3.1.4. Contact Angle

The contact angle was performed to determine the wettability of the scaffolds and is shown in Figure 2d. All scaffolds showed a contact angle of less than 90°. The GC (38.55 ± 1.56°) exhibited the lowest contact angle among the scaffolds. Meanwhile, the CR (64.45 ± 3.75°) has a higher contact angle compared to NC (50.85 ± 7.06°). In comparison, both BNC (48.97 ± 3.68°) and BCR (47.37 ± 1.83°) scaffolds showed no significant differences (*p* < 0.05).

#### 3.1.5. Mechanical Properties

**Figure 3:**
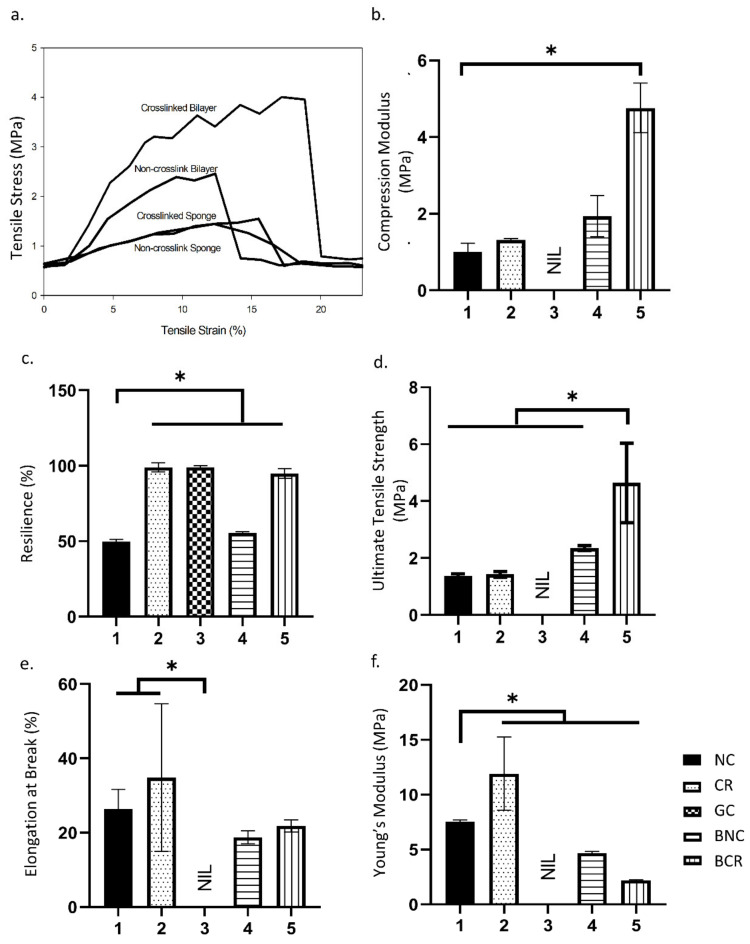
The mechanical characterisation of the scaffolds at room temperature. (**a**) stress vs. strain curve, (**b**) compression test, (**c**) resilience test, (**d**) elongation at break, (**e**) ultimate tensile strength, and (**f**) Young’s modulus. * *p* < 0.05 indicates the siginificant differences of the fabricated scaffolds.

Mechanical strength is vital to withstand the pressure during the implantation of biomatrix at the wound site. The scaffolds showed similar stress vs strain patterns in which stress directly proportional with strain, as seen in Figure 3a. The scaffolds’ compression modulus was performed to determine compressive strength of scaffolds when pressure was asserted, as shown in Figure 3b. The result shown that the bilayer scaffolds were able to withstand pressure better compared to the single layer scaffolds where the compressive moduli of the fabricated scaffolds are 2.22 ± 0.53 MPa, 5.21 ± 0.65 MPa, 1.18 ± 0.23 MPa, and 1.28 ± 0.04 MPa for BNC, BCR, NC, and CR respectively. The scaffolds’ resilience was performed to determine the scaffold’s ability to return to its original shape after the force was applied, as demonstrated in Figure 3c. The resilience of the crosslinked scaffolds was higher compared to the non-crosslinked scaffolds in which the value of NC, CR, GC, BNC, and BCR are 49.70 ± 1.67%, 98.94 ± 2.98%, 99.00 ± 1.00%, 55.60 ± 0.73%, and 94.98 ± 3.20%, respectively. Besides, the stress-strain curve calculated the elongation at break, ultimate tensile strength, and Young’s modulus value. In addition, the elongation at break was performed determining the increasing length of the scaffolds before the break, as stipulated in Figure 3e. The crosslinked scaffolds have a higher elongation at break compared to the non-crosslinked scaffolds. The data for NC, CR, BNC, and BCR are presented as 26.39 ± 5.27%, 34.85 ± 19.85%, 18.78 ± 1.73%, and 21.82 ± 1.66%, respectively. Meanwhile, the ultimate tensile strength in Figure 3d was used to determine the maximum force applied on the bioscaffolds before they break. The BCR needed the highest force to break (4.64 ± 1.40 MPa), indicating that the BCR is the toughest scaffold compared to the other fabricated scaffolds. The ultimate strength of NC, CR, and BNC are 1.37 ± 0.08 MPa, 1.43 ± 1.04 MPa, and 2.36 ± 0.09 MPa, respectively. Young’s modulus was used to determine the elasticity of the scaffolds in reflection of its mechanical strength, as shown in Figure 3f. The mechanical behaviour of the scaffolds can also be determined through Young’s modulus to ensure that the scaffolds will not hinder the patient’s daily activites such as stretching and was also applied during the application of scaffolds into the wound site. A single collagen sponge demonstrated a lower Young’s modulus at 7.56 ± 0.14 MPa compared to the other single composite bioscaffolds at 11.91 ± 3.34 MPa in CR. In comparison, Young’s modulus in bilayer scaffolds was found to be 4.68 ± 0.14 MPa and 2.19 ± 0.06 MPa in non-crosslink and crosslinked bioscaffolds, respectively. The mechanical properties of the scaffolds increased when gelatin/cellulose hydrogel was added. The mechanical properties of the scaffolds were summarised in Figure 3.

#### 3.1.6. Water Vapor Transmission Rate (WVTR)

**Figure 4:**
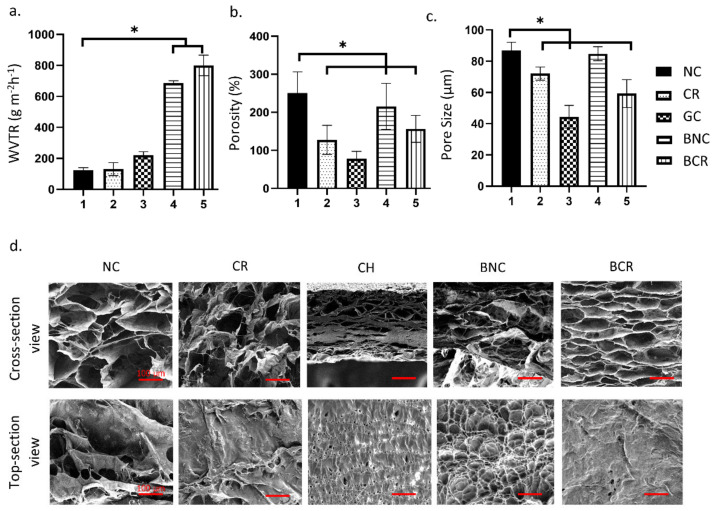
The porosity study of the scaffolds at room temperature. (**a**) The water vapor transmission rate, (**b**) the porosity of the scaffolds, (**c**) the pore size of the scaffolds, and SEM morphology of the scaffolds (**d**) cross-section view of scaffolds and top section view of scaffolds. * *p* < 0.05 indicates the siginificant differences of the fabricated scaffolds.

The WVTR of the scaffolds was measured to assess the rate of water vapour permeated through the bioscaffolds at a specific condition, as demonstrated in Figure 4a. The highest WVTR was recorded for the BCR, resulting in 800.00 ± 65.85 gm^−2^ h^−1^ followed by BNC scaffold with the WVTR of 687.25 ± 14.14 gm^−2^ h^−1^. In contrast, the WVTR for single layer NC, CR, and GC scaffolds were 122.75 ± 17.96, 131.22 ± 41.90, and 220.11 ± 23.94 gm^−2^ h^−1^, respectively. There was a significant increase from NC with BNC and BCR (*p* < 0.05).

#### 3.1.7. Porosity

The porosity percentage of the scaffolds is described in Figure 4b. Most of the samples showed reasonable porosity (>50%) except for the GC (44.44 ± 7.26%). The single-layer NC (86.83 ± 5.26%) showed the highest porosity followed by the BNC (84.83 ± 4.48%). However, the crosslinked scaffolds showed a significant decrease (*p* < 0.05) in both single (72.07 ± 4.22%) and bilayer scaffolds (59.29 ± 8.92%), respectively. The porosities of the scaffolds gradually decreased after crosslinking with 0.1% genipin.

The pore size of the scaffolds decreased after crosslinking with 0.1% genipin (Figure 4c). NC showed to have highest pore size (250.62 ± 55.60 µm) followed by BNC (215.45 ± 62.59 µm). For crosslinked scaffolds, BCR (156.49 ± 35.10 µm) demonstrated a slightly bigger pore size compared to CR (127.71 ± 36.61 µm). The GC (78.63 ± 16.83 µm) also had the smallest pore size compared with other scaffolds to limit the entrance of foreign substances to the wound site. The morphology of the scaffolds was observed under SEM and resulted in a good distribution of porous microstructure (Figure 4d). Some studies reported that the recommended average pore size for skin wound healing is in the range of 100–200 µm [30,31,33].

### 3.2. Chemical Characterisation of Fabricated Biomatrix

#### 3.2.1. Energy Dispersive X-ray Spectrometry (EDX)

**Figure 5:**
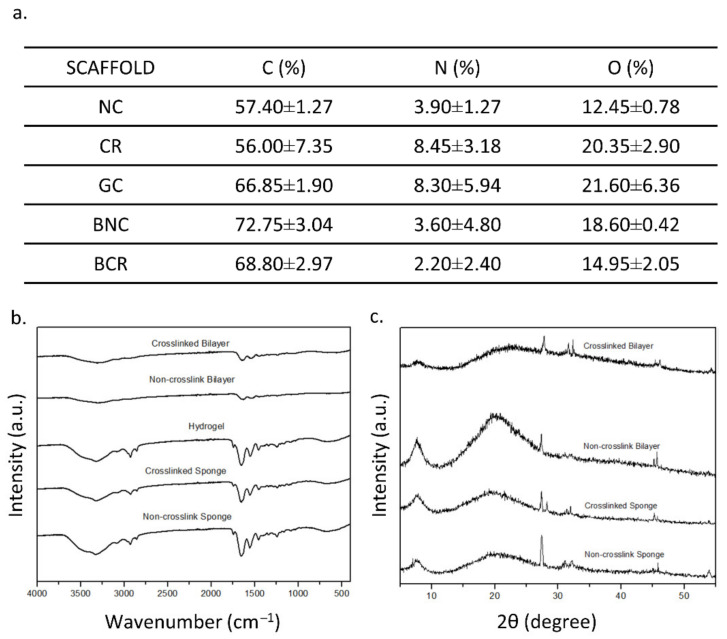
The chemical characteristics of the scaffolds at room temperature. (**a**) The EDX results, (**b**) FTIR results, and (**c**) XRD results of the scaffolds. * *p* < 0.05 indicates the siginificant differences of the fabricated scaffolds.

The EDX was employed to provide the compositional analysis that can be used to detect the homogeneity of the fabricated bioscaffold where element carbon, oxygen, and nitrogen are the main elements in the collagen. The EDX data were summarised in Figure 5a. There are no significant changes in the percentages of element carbon, oxygen and nitrogen in the fabricated scaffolds. The non-crosslink bioscaffolds hold the highest carbon percentage in both single and bilayer, with a value of 57.40 ± 1.27% and 72.75 ± 3.04%, respectively, followed by the gelatin-cellulose hydrogel with a carbon percentage of 66.85 ± 1.90%. In comparison, crosslinked bioscaffolds such as CR and bilayer demonstrated a carbon value of 56.00 ± 7.35% and 68.80 ± 2.97%, respectively. There was no significant difference (*p* > 0.05) in the percentage of nitrogen between NC, CR, GC, BNC, and BCR (3.90 ± 1.27, 8.45 ± 3.18, 3.60 ± 4.80, and 2.20 ± 2.40%, respectively).

#### 3.2.2. Fourier Transform InfraRed (FTIR)

The FTIR spectra of the scaffolds are shown in Figure 5b. The IR spectrum of NC showed the properties of amide A due to the NH stretching (3326.176 cm^−1^), amide B due to CH_2_ asymmetrical stretching (2927.941 cm^−1^), amine I due to NH bending (1652.722 cm^−1^), amine II due to CN stretching (1456.015 cm^−1^), and amine III (1241.952 cm^−1^). A similar spectrum was also observed in the CR. Meanwhile, the bilayer scaffolds captured the spectrum of nanocellulose, referring to the presence of a pyranose ring C-O-C skeletal vibration at 1056.82 cm^−1^. The presence of pyranose ring indicated the presence of nanocellulose in the composite. The bilayer scaffolds also showed a similar collagen type I spectrum with a single-layer sponge.

#### 3.2.3. X-ray Diffraction (XRD)

The purpose of XRD is to determine the crystallographic structure of a material. The XRD patterns of fabricated scaffolds are described in Figure 5c. Briefly, all bioscaffolds showed similar patterns of diffractograms. There are no higher peaks in the X-ray diffractograms, which indicated that the fabricated bioscaffolds were an amorphous phase. The fabricated scaffolds, NC, CR, BNC, and BCR had a high percentage of amorphous, which were 76.87, 82.72, 74.46, and 61.08%, respectively. In addition, there were high broad peaks at 8° and 20° followed by a small peak at 28°, indicating the presence of crystalline powder.

#### 3.2.4. Antibacterial Properties

**Figure 6:**
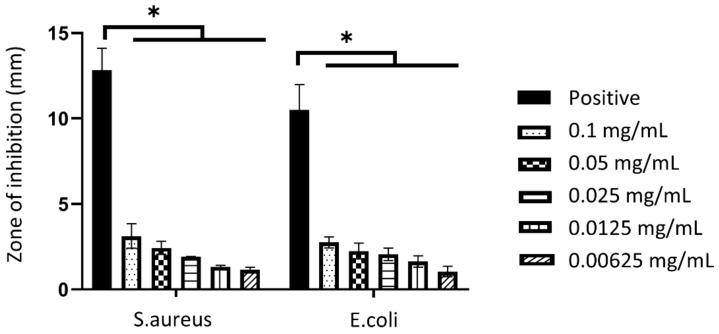
The antibacterial properties of hybrid graphene oxide and silver nanoparticles at 37 °C. * *p* < 0.05 indicates the siginificant differences of the zone of inhibition caused by the nanoparticles.

The disk diffusion assay was carried out to determine the susceptibility of bacteria towards nanoparticles, as shown in Figure 6. The presence of a bacterial inhibition zone indicated that the nanoparticles have antibacterial properties. Gentamicin was used as the positive control on *S. aureus* and *E. coli* with the zone of inhibition of 12.82 ± 1.28 mm and 10.51 ± 1.47 mm, respectively. The biggest zone of inhibition in both *S. aureus* and *E. coli* are seen in the highest concentration of GO-AgNP (0.1 mg/mL), which were 3.12 ± 0.72 mm and 2.75 ± 0.32 mm, respectively. In comparison, the zone of inhibition gradually decreased with the concentration of GO-AgNP (0.05, 0.025, 0.0125, and 0.00625 mg/mL) in *S. aureus*, which was 2.43 ± 0.38 mm, 1.91 ± 0.02 mm, 1.32 ± 0.08 mm, 1.16 ± 0.14 mm, respectively. A similar trend was also seen in *E. coli* for 0.05, 0.025, 0.0125, and 0.00625 mg/mL of GO-AgNP with 2.25 ± 0.46 mm, 2.06 ± 0.37 mm, 1.63 ± 0.33 mm, 1.05 ± 0.30 mm, correspondingly.

### 3.3. Cellular Compatibility

**Figure 7:**
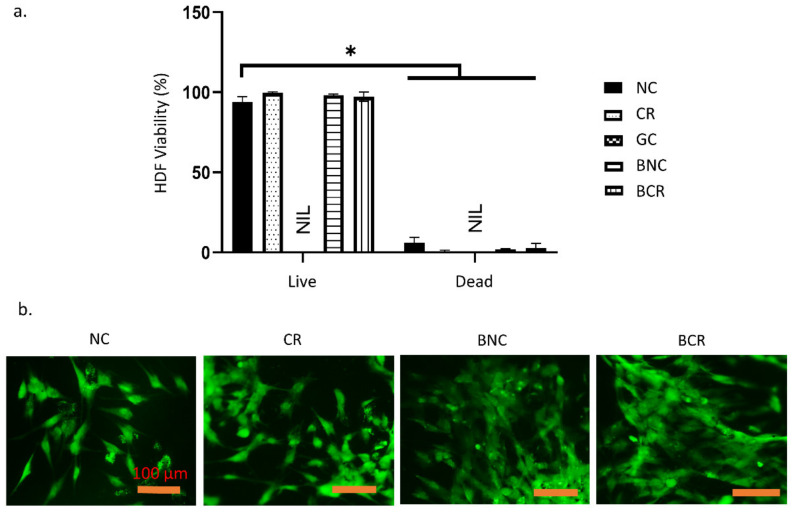
The cellular compatibility of the scaffolds with human dermal fibroblasts after 24 h incubation at 37 °C. (**a**) The cell viability quantification of human dermal fibroblasts using Live and Dead assay and (**b**) the morphology of the human dermal fibroblasts on the fabricated scaffolds, which primarily presented spindle-like cells. * *p* < 0.05 indicates the siginificant differences of the fabricated scaffolds.

Live/dead staining estimated the cell viability of human dermal fibroblasts (HDF) on the scaffolds and was estimated with live/dead staining. The data is presented in Figure 7a,b, which shows the morphologies of the cells in the 3D-bioscaffolds. There was a high ratio of green-stained cells (>90%) on the scaffolds, indicating the presence of live cells. The percentage of live cells was the highest in CR (98.57 ± 1.36%) scaffold then pursued by NC with 98.00 ± 2.14%, BCR 94.83 ± 4.95% and finally in BNC (94.16 ± 8.15%), which has the lowest percentage of live cells. Meanwhile, the BNC showed the highest number of dead cells (5.84 ± 8.15%) compared to the other fabricated scaffolds. There are no significant differences in the study (*p* > 0.05).

## 4. Discussion

A tissue-engineered product is a virtual medical device used to repair and regenerate tissue via scaffolds, cells, and growth factors components [34]. The goal in this study was to maximize the healing capacity and capability with a better gross appearance (less visible scarring) by developing a three-dimensional microstructure that can regenerate and restore the loss of function in the skin. The performances of fabricated bioscaffold are primarily based on their specific properties such as microstructure and interaction towards specific cells to achieve ideal tissue engineering implementation.

In this study, hybrid bilayer scaffolds were successfully fabricated from lyophilisation of ovine tendon collagen-I sponge and cellulose/gelatin hydrogel to produce a heterogeneous porous microstructure and stable bioscaffolds as an ideal acellular skin substitute end-product. The hybrid nanoparticles will be added in future applications as an antimicrobial treatment. The innovative idea behind this biomatrix is to be applied as a one-time application post-implantation acellular skin substitute. Briefly, the collagen materials would slowly degrade inside the skin, followed by tissue regeneration, and the gelatin-cellulose hydrogel would peel off together with the crusted wounds.

The first step in idealising bio-scaffold functionality is primarily to evaluate the physicochemical properties further. It is an unavoidable step to ensure that the output meets the fundamental baseline for cutaneous application. For instance, the fabricated bilayer scaffolds have good water absorption ability, which helps to prevent the accumulation of wound exudates in chronic skin wounds. Another parameter that was prioritised is the in vitro biodegradation, as the current prominent drawback in tissue engineering products is the fast biodegradation of biomaterials post-implantation. The fabricated bilayer scaffolds demonstrated a slow biodegradation rate in an in vitro enzymatic microenvironment at body temperature. The duration for the selected biomaterials in cutaneous wound healing should at least be within 14 days prior to fully becoming degraded, while the implanted site could regenerate the newly formed tissue [35,36]. A previous study performed by Busra and co-researchers (2019) proved that an ovine collagen type I sponge with a mean of 0.01 g/hour is able to support a full wound closure within 14 days in an in vivo model [37]. The stability of the scaffolds was controlled by the crosslinking reaction with a chemical crosslinker—genipin. The reaction of collagen and genipin produces a strong covalent bond, which stabilises the scaffolds and thus slows down the degradation of the scaffolds.

**Figure 8:**
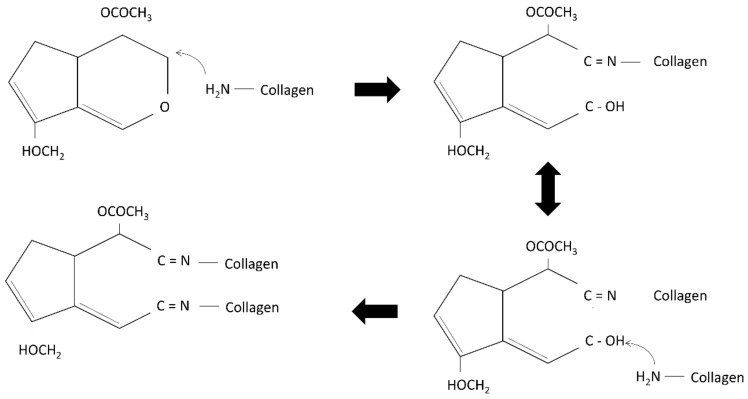
The schematic structure of the crosslinking mechanism between collagen and genipin. The figures was adapted from Riacci et. al., 2021 [44] and was licensed under Creative Commons CC BY 4.0.

In the current study, the crosslinked bilayered biomatrix was successfully developed to mimic the mechanical properties of native skin and proved to be suitable for skin application. In addition, the bilayer scaffolds also showed high mechanical properties similar to the mechanical strength of native tissue to avoid friction due to the stress-shielding mechanism [38]. At the same time, the achieved Young’s modulus is in the range 4.6 to 20.0 MPa [39]. The main reason contributing to the scaffolds’ high mechanical properties is most likely due to the addition of genipin as a natural crosslinker that affects the microstructure stability of the fabricated scaffolds as shown in Figure 8 [40,41]. Other essential features of the scaffolds are to support the transfusion of gases and water vapour to prevent excessive dryness in the wound bed. The developed bilayer scaffolds can achieve the minimum requirement of WVTR, as they are within the range of commercially available acellular skin substitutes [25,26]. The FTIR analysis showed typical amines I, II, and III absorbance peaks at an infrared wavenumber from 1660 cm^−1^ to 1241 cm^−1^. Meanwhile, the presence of pyranose ring was detected at a wavenumber of 1056 cm^−1,^ indicating the presence of cellulose in the bilayered bioscaffolds [42]. A broad peak in the XRD resulted from the amorphous properties, indicating the native origin of the fabricated bioscaffolds. Besides, at 8°, a high peak indicates the triple-helix bond of collagen, supporting the FTIR findings [43]. Both physicochemical and mechanical findings indicated that the fabricated scaffolds preserve their original native properties while improving the mechanical strength, resembling native skin tissue microstructure.

Furthermore, the current scenario in chronic wound healing is facing an extreme challenge in order to combat bacterial infection, which is causing unsuccessful post-transplantation of tissue engineering products [45,46]. Therefore, this study provides a functional biomaterial design with the presence of GO-AgNP, which acts as an antibacterial agent in the fabricated biomatrix. The nanoparticles, however, were embedded in the cellulose/gelatin hydrogel to prevent the entrance of nanoparticles into the human blood system, particularly to avoid the long-term effect of nanoparticles in the body [47,48]. Besides, silver nanoparticles have great potential as antibacterial agents, as they possess a broad spectrum of antibacterial, antifungal, and antiviral properties [49]. Meanwhile, graphene oxide has been proven to prevent the aggregation of silver nanoparticles and accelerate the efficiency of silver nanoparticles’ performance as antibacterial agents [50,51]. The promising positive results of hybrid nanoparticles presenting antibacterial properties enhance the synergistic effect of currently fabricated hybrid bioscaffolds for future cutaneous wound injury.

Even though the integration of GO-AgNP with fabricated bilayer bioscaffolds has been successfully developed, cellular compatibility would be another challenge as a tissue engineering product. The wrong selection of an antibacterial agent could affect the homing cells’ viability post-implantation [52], and thus the hybrid bioscaffold’s biocompatibility is a key factor to determine whether the scaffolds are suitable for tissue engineering application [53]. The fabricated bilayer scaffolds showed no cytotoxic effect on human dermal fibroblasts. Previous studies have proven that collagen type I and genipin promoted good cell proliferation and attachment in various cells [31,54]. At the same time, the scaffolds have a relatively high porous structure, which contributed to cell adhesion, growth, and proliferation, which could synergistically enhance tissue regeneration [55]. However, further in vitro and in vivo studies are needed to escalate the evaluation of the efficiency of the fabricated bioscaffolds as functional acellular skin substitutes in the near future.

## 5. Conclusions

The fabrication of bilayer scaffolds has been successfully developed using the freeze-drying method and presented promising physicochemical and mechanical properties as an acellular skin substitute. The presence of genipin into the hybrid bilayer biomatrix increased its mechanical strength and micro-stability. The amorphous properties of the scaffolds have made it possible to apply them in an irregular shape in deep irregular wounds such as a diabetic ulcer or traumatic wound. The fabricated biomatrix is also biocompatible with the human dermal fibroblasts, although it has to be incorporated with silver nanoparticles to act as an antibacterial agent. Nevertheless, further studies should be performed to verify the functionality of the fabricated biomatrix as wound care products that are able to support the dynamic process of wound healing.

## Data Availability

Not Applicable.
